# Chimeric HIV-1 Envelope Glycoproteins with Potent Intrinsic Granulocyte-Macrophage Colony-Stimulating Factor (GM-CSF) Activity*

**DOI:** 10.1371/journal.pone.0060126

**Published:** 2013-04-02

**Authors:** Gözde Isik, Thijs van Montfort, Maikel Boot, Viviana Cobos Jiménez, Neeltje A. Kootstra, Rogier W. Sanders

**Affiliations:** 1 Department of Medical Microbiology, Laboratory of Experimental Virology, Center for Infection and Immunity Amsterdam, Academic Medical Center, University of Amsterdam, Amsterdam, The Netherlands; 2 Department of Experimental Immunology, Sanquin Research, Landsteiner Laboratory, Center for Infection and Immunity Amsterdam, Academic Medical Center, University of Amsterdam, Amsterdam, The Netherlands; 3 Department of Microbiology and Immunology, Weill Medical College of Cornell University, New York, United States of America; University of California San Francisco, United States of America

## Abstract

HIV-1 acquisition can be prevented by broadly neutralizing antibodies (BrNAbs) that target the envelope glycoprotein complex (Env). An ideal vaccine should therefore be able to induce BrNAbs that can provide immunity over a prolonged period of time, but the low intrinsic immunogenicity of HIV-1 Env makes the elicitation of such BrNAbs challenging. Co-stimulatory molecules can increase the immunogenicity of Env and we have engineered a soluble chimeric Env trimer with an embedded granulocyte-macrophage colony-stimulating factor (GM-CSF) domain. This chimeric molecule induced enhanced B and helper T cell responses in mice compared to Env without GM-CSF. We studied whether we could optimize the activity of the embedded GM-CSF as well as the antigenic structure of the Env component of the chimeric molecule. We assessed the effect of truncating GM-CSF, removing glycosylation-sites in GM-CSF, and adjusting the linker length between GM-CSF and Env. One of our designed Env_GM-CSF_ chimeras improved GM-CSF-dependent cell proliferation by 6-fold, reaching the same activity as soluble recombinant GM-CSF. In addition, we incorporated GM-CSF into a cleavable Env trimer and found that insertion of GM-CSF did not compromise Env cleavage, while Env cleavage did not compromise GM-CSF activity. Importantly, these optimized Env_GM-CSF_ proteins were able to differentiate human monocytes into cells with a macrophage-like phenotype. Chimeric Env_GM-CSF_ should be useful for improving humoral immunity against HIV-1 and these studies should inform the design of other chimeric proteins.

## Introduction

Despite 20 years of research, a protective vaccine against HIV-1 is still elusive. Generating protective immunity against HIV-1 has proven to be extremely challenging and vaccines tested in several large-scale clinical trials induced no or very modest protection. An ideal HIV-1 vaccine should activate both the humoral and cellular arms of the immune system and the vaccines designed to stimulate only one arm have failed. The recent RV144 trial, designed to generate both B and T cell responses by combining an HIV-1 protein expressing pox-virus prime with a recombinant gp120 boost, yielded a modest 31.2% efficacy, providing hope that potent vaccine protection may eventually be achievable [1].

Broadly neutralizing Abs (BrNAbs) can provide sterile immunity in non-human primates when administered passively, but no HIV-1 vaccine tested to date has been able to induce such BrNAbs. The HIV-1 envelope glycoprotein complex (Env), located on the outside of the virus particle, is the only relevant protein for the induction of BrNAbs, but a number of structural Env properties limit the induction of BrNAbs. Env contains surface loops that are highly variable in sequence between different HIV-1 isolates and these variable loops shield the conserved BrNAb targets [2]–[7]. Moreover, the HIV-1 Env is heavily glycosylated and the “glycan shield” protects underlying protein domains from Ab recognition, although the recent identification of a number of glycan-dependent BrNAbs suggests that the glycan shield itself can be targeted by BrNAbs [8]–[11]. The presence of *N*-linked oligomannose glycans on Env can also facilitate suppression of dendritic cell-mediated immune activation and limit the induction of Ab responses [12]–[17]. Shedding of monomeric gp120 from Env trimers or expression of nonfunctional Env forms on the surface of HIV particles may divert the immune response to immune-dominant epitopes that are not exposed on functional Env trimers and thus may act as immune decoys [16], [18]. Processing of the Env glycoprotein precursor into the gp120 and gp41 subunits also affects antigenicity; compared to uncleaved trimers, cleaved trimers are better mimics of the native Env trimer on virus particles and they interact more efficiently with BrNAbs than with non-neutralizing Abs [19]–[22]. Whether cleavage also affects Env trimer immunogenicity is currently unresolved.

Env’s various defense mechanisms to evade humoral immunity forces us to search for unorthodox approaches to improve Env immunogenicity. We have constructed a chimeric Env protein, termed Env_GM-CSF_, in which the first and second variable domains (V1V2) were replaced by GM-CSF, with the aim of improving immune responses against Env [23]. GM-CSF acts as a survival, proliferation, and differentiation factor for several hematopoietic precursor cell populations [24]–[26]. It is secreted during inflammation to prevent apoptosis and to stimulate differentiation and proliferation of various immune cells [27], including antigen presenting cells (APCs) [28]. The covalent conjugation of Env and GM-CSF ensures that only immune cells that see the antigen are activated by the GM-CSF component. Immunization studies in mice showed that Env_GM-CSF_ induced improved antibody and T helper responses against Env compared to unconjugated Env [23]. Although the chimeric Env_GM-CSF_ molecule was more immunogenic compared to Env_wt_, *in vitro* studies indicated that the GM-CSF domain within Env_GM-CSF_ was not as active as recombinant GM-CSF. Moreover, the insertion of GM-CSF in the V1V2 region resulted in subtle perturbation of the antigenicity of the CD4 binding site (CD4BS) and CD4-induced (CD4i) epitopes, which are positioned in close proximity to the inserted GM-CSF molecule.

In this study, we aimed to improve the GM-CSF activity and Env conformation of the Env_GM-CSF_ immunogen by the following approaches: i) inserting of smaller GM-CSF domains; ii) modifying the disulfide bonded architecture of Env_GM-CSF_; iii) removing the *N*-linked glycosylation sites of the GM-CSF domain; iv) optimizing the flexible linkers between the Env and GM-CSF domains; and v) embedding GM-CSF within cleaved Env trimers. The results show that the GM-CSF activity of Env_GM-CSF_ could be increased by 6-fold by extending the peptide linker between the GM-CSF and the Env. Interestingly, rendering Env_GM-CSF_ competent to proteolytic cleavage between gp120 and gp41 further improved the GM-CSF activity. The improved Env_GM-CSF_ variants drove the differentiation of primary monocytes into macrophages, testifying that these chimeric molecules are able to activate human immune cells. These optimized chimeric Env_GM-CSF_ variants should be useful HIV-1 vaccine candidates. Furthermore, these studies should assist the design of other chimeric molecules aimed at improving the immunogenicity of HIV-1 Env.

## Results

### Truncation of GM-CSF Reduces GM-CSF Activity of Chimeric Env_GM-CSF_

We observed that replacement of the V1V2 domain by human GM-CSF had a minor effect on the exposure of the CD4 binding site (CD4BS), and decreased the exposure of CD4-induced (CD4i) antibody epitopes upon CD4 binding [23]. One hypothesis is that these effects could be caused by the insertion of the larger GM-CSF molecule plus linkers (in total 120 amino acids) at the expense of the smaller JRFL V1V2 domain (65 amino acids).

**Figure 1:**
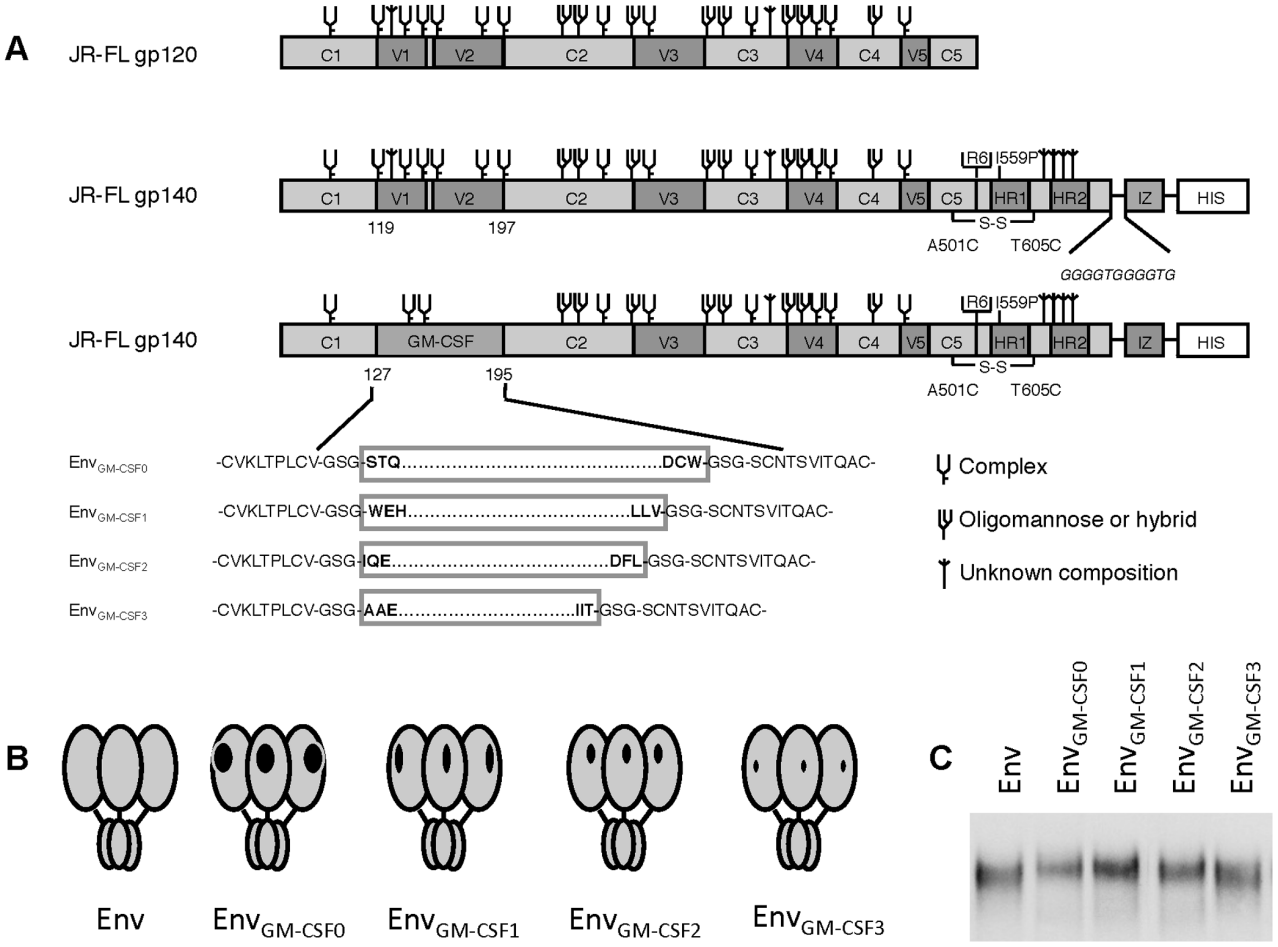
Schematics and expression of the Env_wt_, Env_GM-CSF_ and truncated Env_GM-CSF_ variants Linear (A) and cartoon (B) representation of the original and truncated Env_GM-CSF_ proteins. The clade B JRFL gp140 protein (amino acids 31 to 681) contains several modifications for stabilization that have been previously described (see Materials and Methods). Codon optimized sequences encoding human GM-CSF1 (amino acids 13 to 118), GM-CSF2 (amino acids 19 to 114), and GM-CSF3 (amino acids 33 to 112) were inserted to the V1V2 domain of gp140. Env subdomains are indicated: 5 conserved domains (C1–C5); 5 variable domains (V1–V5); heptad repeats 1 and 2 (HR1, HR2); the trimerization domain (IZ) and the histidine tag, comprised of 8 histidine amino acids (HIS). The glycan assignments in Env are based on previous studies using gp120 [60]–[62]. The composition of GM-CSF *N*-glycans is reported in ref [63]. C. Chimeric Env_GMCSF1/2/3_ proteins expressed transiently in 293T cells were analyzed in reducing SDS-PAGE analysis followed by western blot.

We studied whether we could increase the recognition of the CD4BS and in particular the CD4i epitopes within the Env_GM-CSF_ molecule by trimming the N- and C- terminal ends of GM-CSF molecule while maintaining the GM-CSF core that is essential for receptor binding (Fig. 1A&1B). The GM-CSF truncations were designed such that the truncated N- and C- termini were in relatively close proximity to facilitate grafting onto the V1V2 stem. The truncation variants were termed Env_GM-CSF1_, containing the GM-CSF amino acids 13 to 118; Env_GM-CSF2_ (amino acids 19 to 114); and Env_GM-CSF3_ (amino acids 33 to 112) (Fig. 1A). The predicted distances between the N- and C- terminal residues of GM-CSF1, GM-CSF2 and GM-CSF3 were 16.88 Å, 6.65 Å and 11.78 Å, respectively, compared to 12.57 Å for the original Env_GM-CSF_ construct, here termed Env_GM-CSF0_. All Env trimers with truncated GM-CSF proteins were expressed similarly (Fig. 1C).

**Figure 2:**
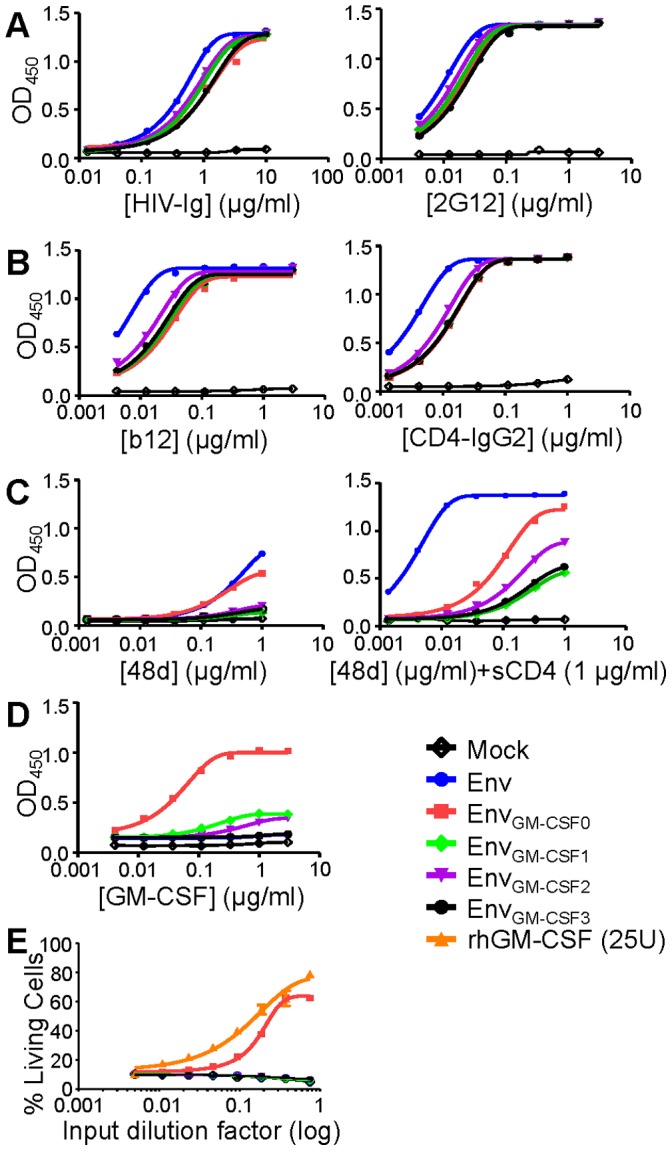
Antigenicity and activity of truncated Env_GM-CSF_ variants. ELISA reactivity of Env_GM-CSF1/2/3_ with HIV-Ig and 2G12 (A); b12 and CD4-IgG2 (B); and 48d (CD4i) in the absence and presence of sCD4 (C). Culture supernatant from mock transfected 293T cells was used as a negative control. D. Recognition of Env_GM-CSF1/2/3_ by the monoclonal conformational GM-CSF antibody (clone 30-4). All ELISA results are representative for at least three independent experiments using proteins derived from three independent transfections. E. Survival of TF-1 cells in response to either culture supernatant containing Env_GM-CSF0/1/2/3_, Env_wt_ or rhGM-CSF. The percentages of living cells after 5 days of culture in a volume of 50 µl are given. Culture supernatant from mock transfected 293T cells was used as a negative control and the values were deducted from the test values.

We evaluated the antigenic structure and conformation of the different Env_GM-CSF_ chimeras by ELISA. Binding of polyclonal Ig from pooled HIV-positive sera (HIV-Ig) was comparable for all Env_GM-CSF_ variants (Fig. 2A). The slightly better binding of HIV-Ig to the Env_wt_ is probably caused by Abs that can bind to the V1V2 domain that is only present in Env_wt_. The 2G12 mAb, which binds to oligomannose *N-*glycans on the outer domain of gp120, bound equally well to the Env_GM-CSF_ variants, but slightly better to Env_wt_. The binding of the CD4BS mAb b12 and the CD4 receptor mimic CD4-IgG2 was affected by the introduction of GM-CSF and this was similar for the smaller Env_GM-CSF_ variants (Fig. 2B). The CD4i mAb 48d did not bind efficiently to Env_wt_ or to the Env_GM-CSF_ variants. In the presence of CD4, 48d bound efficiently to Env_wt_, but the induction of 48d binding to the Env_GM-CSF_ was limited (Fig. 2C). These results indicate that regardless of the size of the inserted GM-CSF domain into the V1V2 region, CD4-induced conformational changes occur inefficiently, suggesting that properties intrinsic to the V1V2 sequence are required for CD4-induced conformational changes (see discussion section).

We next determined the binding of a conformational GM-CSF Ab to the chimeric Env_GM-CSF_ variants. The conformational GM-CSF mAb (30-4) did not bind to Env_wt_, but interacted efficiently with Env_hGM-CSF0_ (Fig. 2D). The binding the GM-CSF mAb was severely affected for the smaller Env_GM-CSF1_ and Env_GM-CSF2_ chimeras, and completely abrogated for the smallest Env_GM-CSF3_ molecule, indicating that the epitope for this mAb is destroyed or inaccessible on the truncated chimeras.

To test the activity of GM-CSF in the Env_GM-CSF_ chimeras we performed survival assays using GM-CSF dependent myeloid leukaemia cells (TF-1 cells). TF-1 cells were cultured in the presence of the various Env_GM-CSF_ variants, and the number of living cells after three days of culturing was determined by FACS flow cytometry, as an indication of GM-CSF activity. As controls we used soluble recombinant GM-CSF (rhGM-CSF), the original Env_GM-CSF_ chimera, Env_wt_ and mock medium. rhGM-CSF and the original Env_hGM-CSF0_ chimera were able to induce survival of TF-1 cells, but none of the three truncated Env_GM-CSF_ variants were able to do so (Fig. 2E), indicating that the truncated amino acids are essential for GM-CSF function. In summary, insertion of smaller GM-CSF domains into the V1V2 loop of Env did not improve Env antigenicity and lead to loss of GM-CSF function.

**Figure 3:**
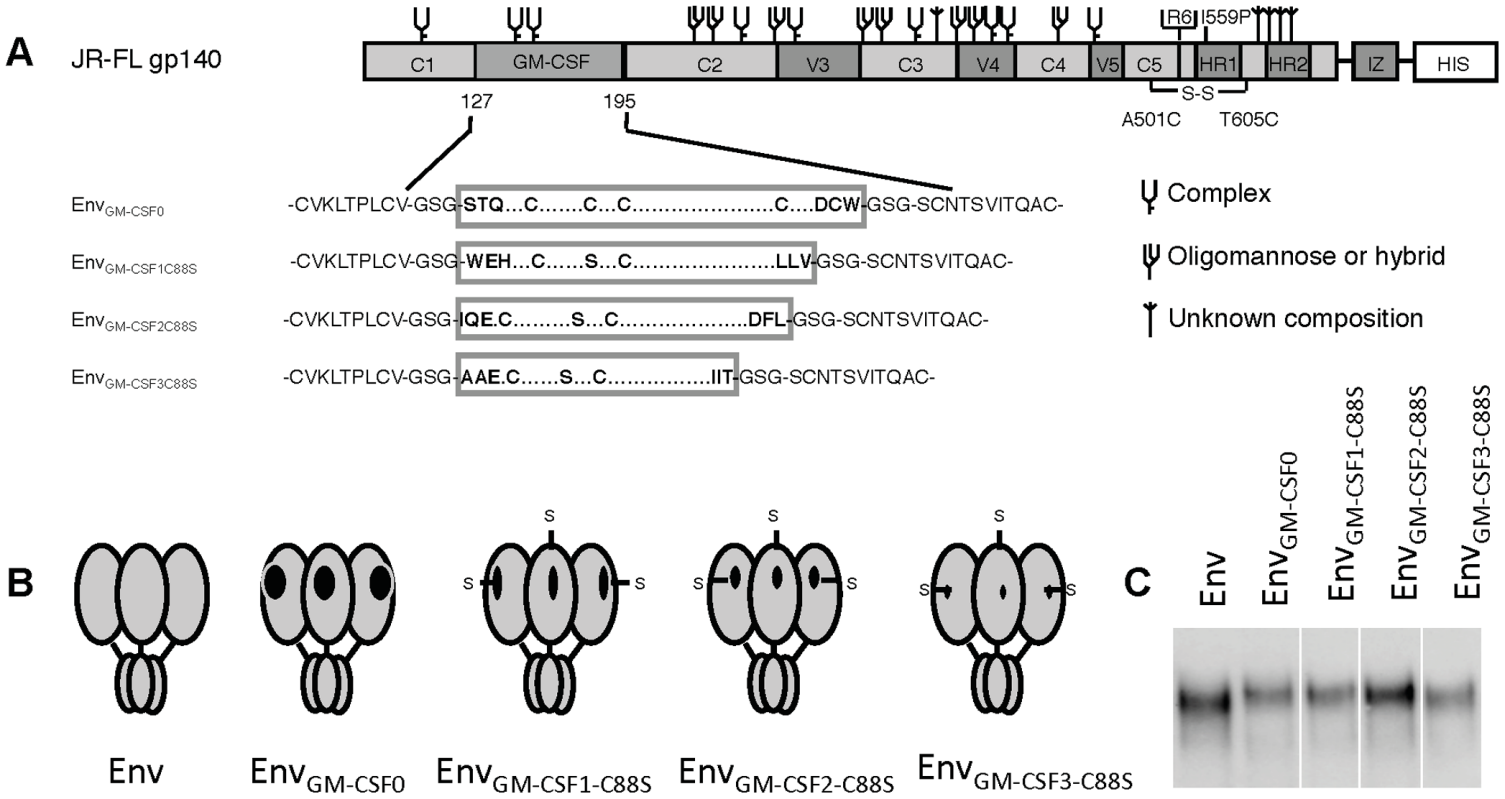
Schematics and expression of truncated and cysteine modified Env_GM-CSF_ variants. Linear (A) and cartoon (B) rendering of the Env_GM-CSF_ and Env_GM-CSF1/2/3-C88S_ constructs. C. The resulting Env_GM-CSF1-C88S_, Env_GM-CSF2-C88S_, Env_GM-CSF3-C88S_ chimeric glycoproteins expressed transiently in 293T cells, were analyzed in reducing SDS-PAGE analysis followed by western blot.

**Figure 4:**
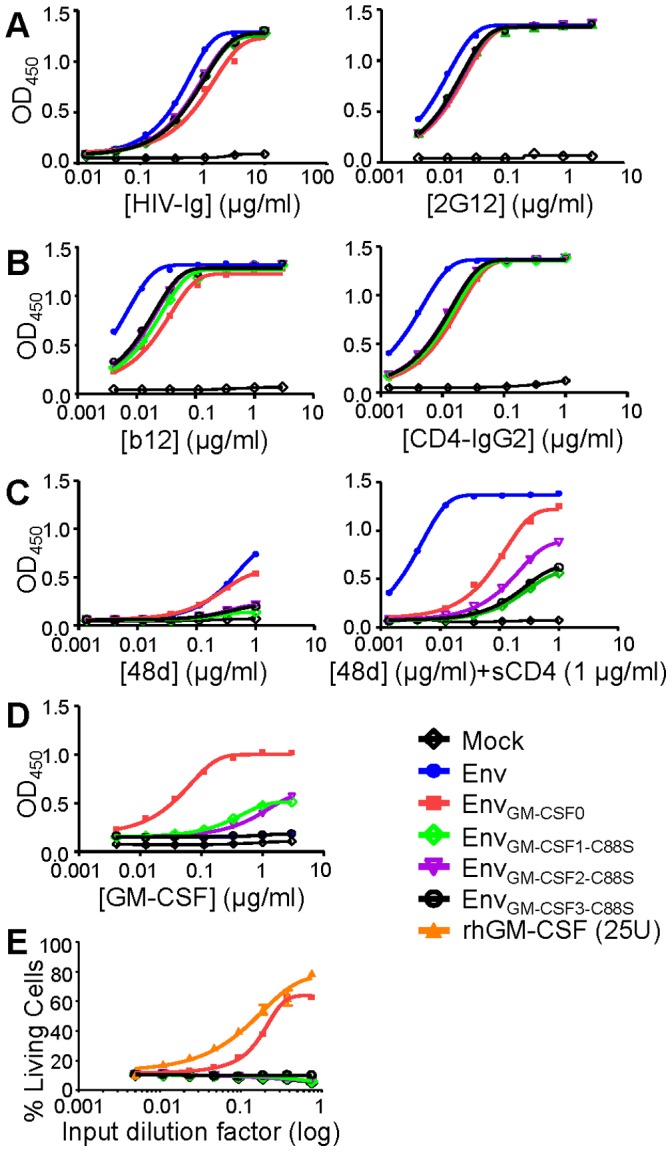
Antigenicity and activity of truncated and cysteine modified Env_GM-CSF_ variants. Recognition of Env_GM-CSF1/2/3-C88S_ by HIV-Ig and 2G12 (A); b12 and CD4-IgG2 (B); and 48d (CD4i) in the absence and presence of sCD4 (C). Culture supernatant from mock transfected 293T cells was used as a negative control. D. Recognition of Env_GMCSF1/2/3-C88S_ by the GM-CSF antibody (clone 30-4). All ELISA results are representative for at least three independent experiments using proteins derived from three independent transfections. E. Survival of TF-1 cells in response to culture supernatant containing Env_GM-CSF0/1/2/3-C88S_, Env_wt_ or rhGM-CSF.

GM-CSF has two disulfide bridges between cysteine residues at positions 54–96 and 88–121 and especially the cysteine bond between residue 54 and 96 is critical for biological activity [29]. Our truncated smaller Env_GM-CSF_ variants lost the cysteine at position 121 in GM-CSF, leaving cysteine 88 unpaired. As a result, the unpaired cysteine 88 could interfere with the formation of a disulfide bridge between cysteine 54 and 96 within GM-CSF and/or with other cysteine bridges within Env [30]. We therefore mutated the cysteine residue at position 88 in GM-CSF to a serine (C88S; Fig. 3A&3B). The resulting Env_GM-CSF1-C88S_, Env_GM-CSF2-C88S_, Env_GM-CSF3-C88S_ chimeric glycoproteins were expressed efficiently (Fig. 3C). Binding of HIV-Ig and 2G12 to the smaller Env_GM-CSF1/2/3-C88S_ was similar compared to Env_wt_ and Env_GM-CSF0_, while the binding of b12 and CD4-IgG2 was again reduced (Fig. 4A&4B). The C88S substitution did not enhance the induction of conformational changes by CD4 as measured by 48d binding, nor did it improve binding of the GM-CSF mAb (Fig. 4C&4D). Moreover, results of the TF-1 assay showed that none of the Env_GM-CSF1/2/3-C88S_ variants were capable to stimulate cell survival (Fig. 4E). Thus, the C88S substitution did not improve the truncated Env_GM-CSF_ variants.

### N-glycosylation at Asp-47 is Essential for GM-CSF Activity of Chimeric Env_GM-CSF_

**Figure 5:**
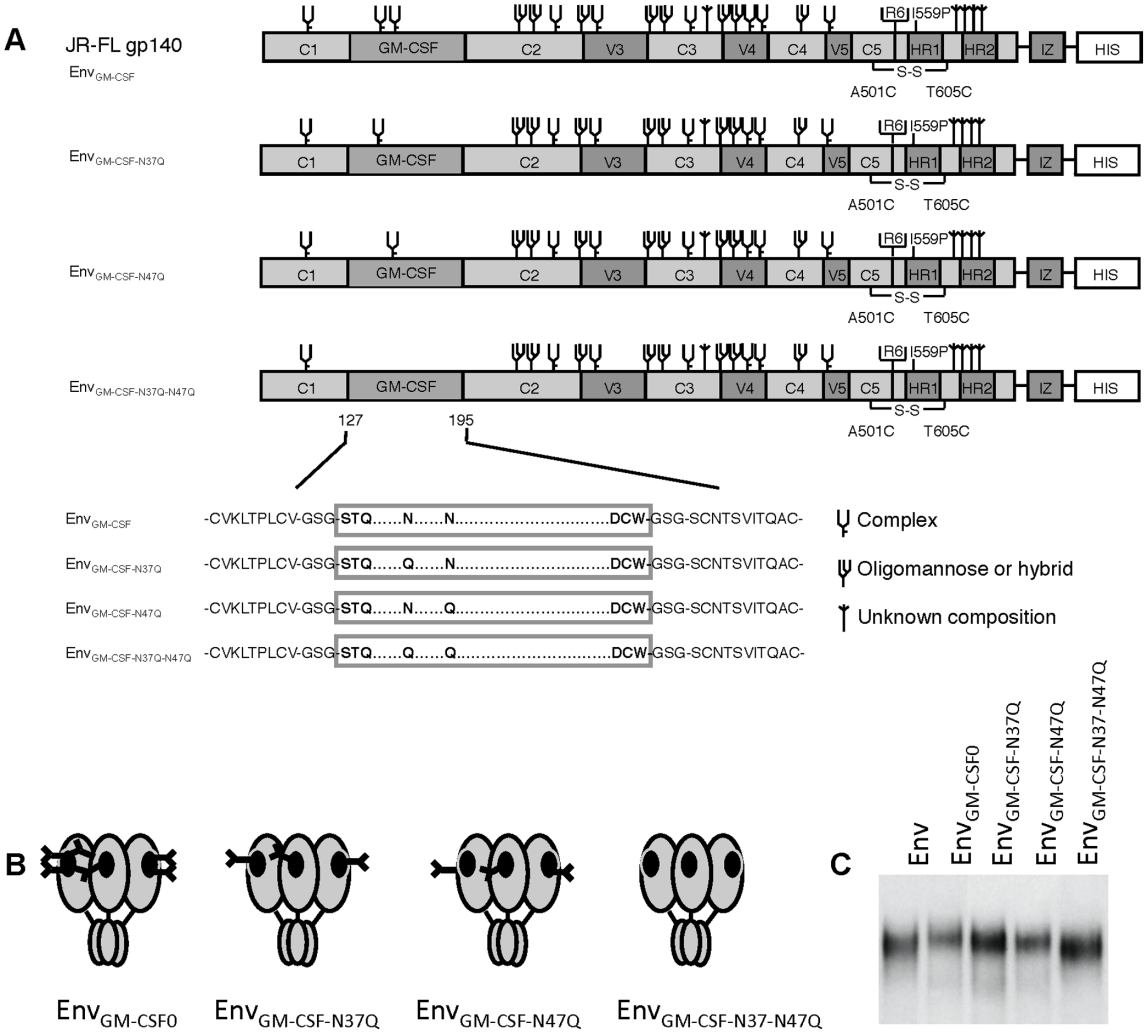
Schematics and expression of Env_GM-CSF_ glycan mutants. Linear (A) and cartoon (B) presentation of Env_GM-CSF-N37Q_, Env_GM-CSFN47Q_ and Env_GM-CSF-N37Q-N47Q_ variants. C. Expression of chimeric Env_GM-CSF-N37Q_, Env_GM-CSF-N47Q_, Env_GM-CSF-N37Q-N47Q_ proteins.

*E. coli* produced rhGM-CSF is not glycosylated, while our Env_GM-CSF_ chimeras produced in human 293T cells can be glycosylated on potential *N*-linked glycan motifs (NxT/NxS) at positions 37 and 47. Previous studies have shown that the functional activity of GM-CSF is increased when the *N*-linked glycans are removed, which could explain the higher activity of rhGM-CSF in our TF-1 stimulation assay [31]. We therefore reasoned that the presence of one or two glycans at the potential *N*-linked glycosylation sites at positions 37 and 47 in GM-CSF might interfere with GM-CSF activity. We aimed to increase the potency of Env_GM-CSF_ by removing one or both glycans by substituting the asparagines for glutamines (N37Q and N47Q) resulting in Env_GM-CSF-N37Q_, Env_GM-CSF-N47Q_ and the double mutant Env_GM-CSF-N37Q-N47Q_ (Fig. 5A). Expression of the Env_GM-CSF_ variants was similar to Env_wt_ and the original Env_GM-CSF_ molecule (Fig. 5B), indicating that the loss of the potential glycosylation site in GM-CSF does not affect the secretion of Env_GM-CSF_. The N37Q and N47Q Env_GM-CSF_ variants migrated slightly faster through the gel compared to the original Env_GM-CSF_ variants, while the double mutant Env_GM-CSF-N37Q-N47Q_ migrated the fastest, indicating that both potential glycosylation sites are occupied by glycans in Env_GM-CSF_ (Fig. 5C).

**Figure 6:**
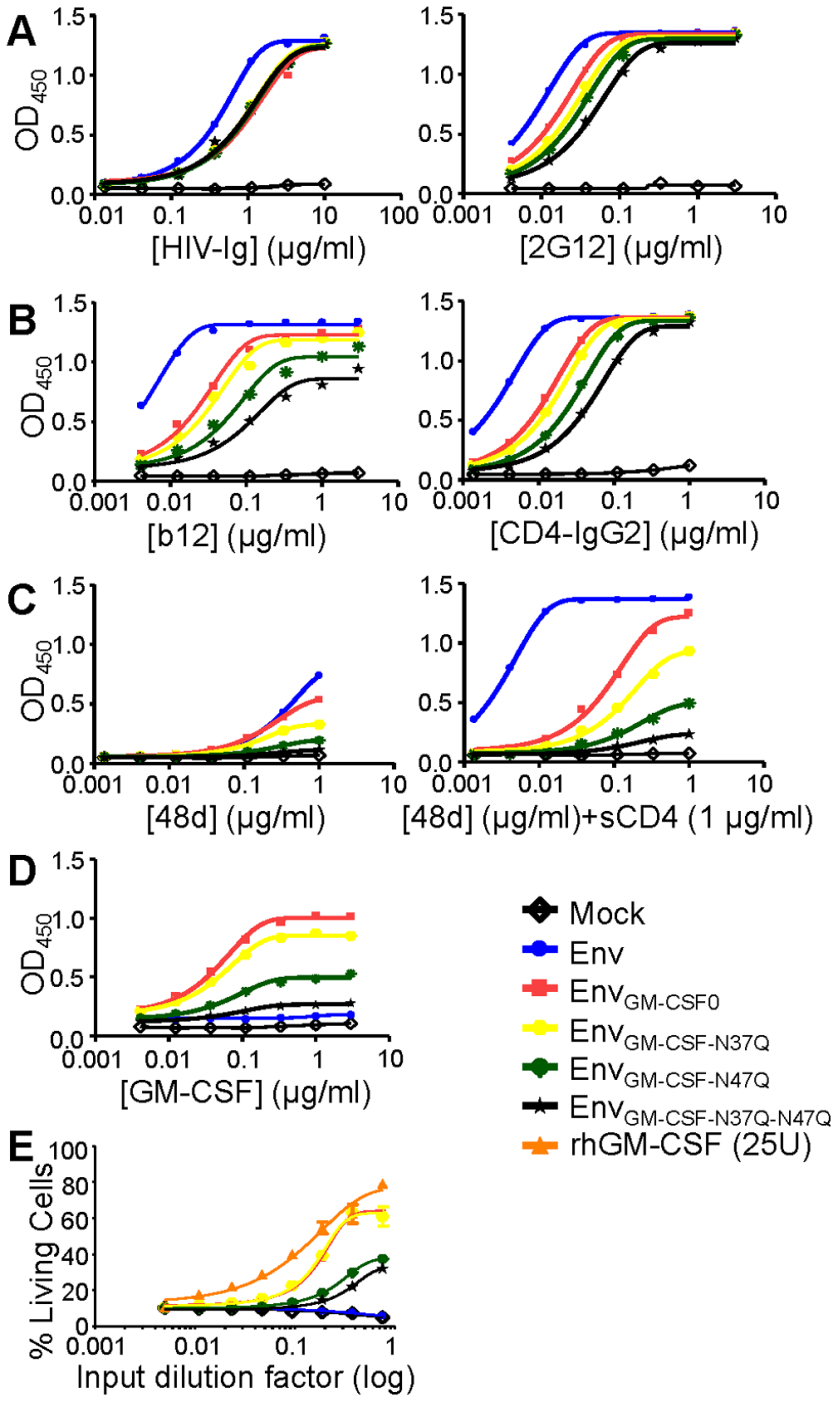
Antigenicity and activity of Env_GM-CSF_ glycan variants. Recognition of Chimeric Env_GMCSF-N37Q_, Env_GM-CSF-N47Q_, Env_GM-CSF-N37Q-N47Q_ by HIV-Ig and 2G12 (A); b12 and CD4-IgG2 (B); and 48d (CD4i) in the absence and presence of sCD4 (C). Culture supernatant from mock transfected 293T cells was used as a negative control. D. Recognition of Env_GMCSF-N37Q_, Env_GM-CSF-N47Q_, Env_GM-CSF-N37Q-N47Q_ by the GMCSF antibody (clone 30-4). All ELISA results are representative for at least three independent experiments using proteins derived from three independent transfections. E. Survival of TF-1 cells in response to culture supernatant containing Env_GM-CSF_, Env_GM-CSF_ variants or controls.

HIV-Ig binding was similar for all Env_GM-CSF_ variants. The glycan-dependent 2G12 mAb bound less efficiently to mutant Env_GM-CSF_ variants, with the lowest binding to the double Env_GM-CSF-N37Q-N47Q_ mutant (Fig. 6A). Binding of b12, CD4-IgG2, and 48d to the Env_GM-CSF_ glycan mutants was decreased in comparison with the fully glycosylated Env_GM-CSF_ chimera (Fig. 6B&6C). The binding of the GM-CSF mAb was slightly affected by the removal of the N-linked glycan at position 37 in the GM-CSF domain, while the removal of the glycan at position 47 dramatically reduced the binding of the GM-CSF mAb (Fig. 6D). Consistent with the GM-CSF mAb binding data, but in contrast to what we anticipated based on a previous work [31], we observed a strong decrease in GM-CSF activity with the Env_GM-CSF_ variant lacking the glycan at position 47. Removal of the glycan at position 37 slightly reduced the GM-CSF activity (Fig. 6E). Thus, while the lack of *N*-linked glycans is not important for the activity of rhGM-CSF expressed in *E.coli*, the glycan at position 47 of GM-CSF is essential for proper folding and/or activity when embedded within HIV-1 Env and expressed in 293T cells.

### Spacing between the GM-CSF and Env Domains Improves GM-CSF Activity

**Figure 7:**
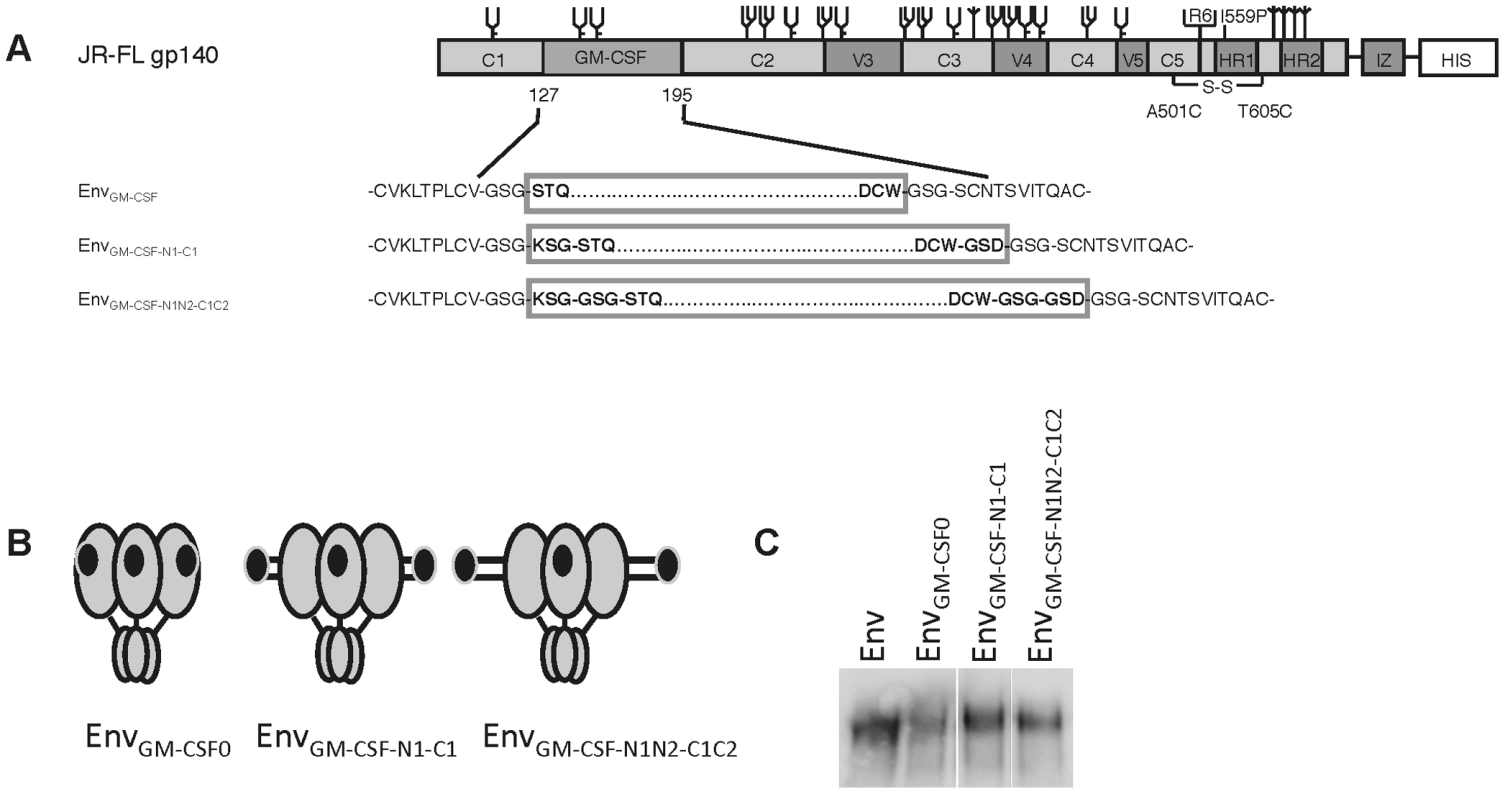
Schematic and expression of the Env_GM-CSF_ with extended linkers. Linear (A) and cartoon (B) rendering of the Env, Env_GM-CSF_, Env_GMCSF-N1-C1_ and Env_GMCSF-N1N2-C1C2_. Env_GM-CSF-N1-C1_ and Env_GM-CSF-N1N2-C1C2_ were created by symmetrical extension of linker regions between Env and GM-CSF with three (KSG-GSD) or six (KSGGSG-GSGGSD) additional amino acids. C. Expression of Env_wt_, chimeric Env_GMCSF-N1-C1_ and Env_GMCSF-N1N2-C1C2_ assessed by SDS-PAGE followed by western blot.

Activity of GM-CSF is mediated by formation of a dodecameric complex with the GM-CSF receptor, consisting of four GM-CSF molecules bound to a dimeric GM-CSF receptor complex containing two alpha and two beta GM-CSF receptor subunits. We hypothesized that three GM-CSF domains embedded within an Env_GM-CSF_ trimer could be inefficient in forming such complexes because of steric clashes with Env domains. We therefore extended the linker region between the Env and the GM-CSF subdomain to allow for more conformational and steric freedom. The linker regions, that originally consisted of three amino acids (Gly-Ser-Gly) on either side of GM-CSF, were extended with three or six amino acids at both termini. The new linkers contained a positively charged lysine (K) in the N-terminal amino acid stretch and a negatively charged aspartic acid (D) on the opposite side in the C-terminal stretch to allow for stability by formation of a salt bridge. The inserted linkers were termed N1 (Lys-Ser-Gly) and N2 (Gly-Ser-Gly) at the N-terminus of GM-CSF; and C1 (Gly-Ser-Gly), C2 (Gly-Ser-Asp) at the C-terminus (Fig. 7A&7B). The expression of Env_GM-CSF-N1-C1_ and Env_GM-CSF-N1N2-C1C2_ was comparable to that of Env_GM-CSF0_ (Fig. 7C).

**Figure 8:**
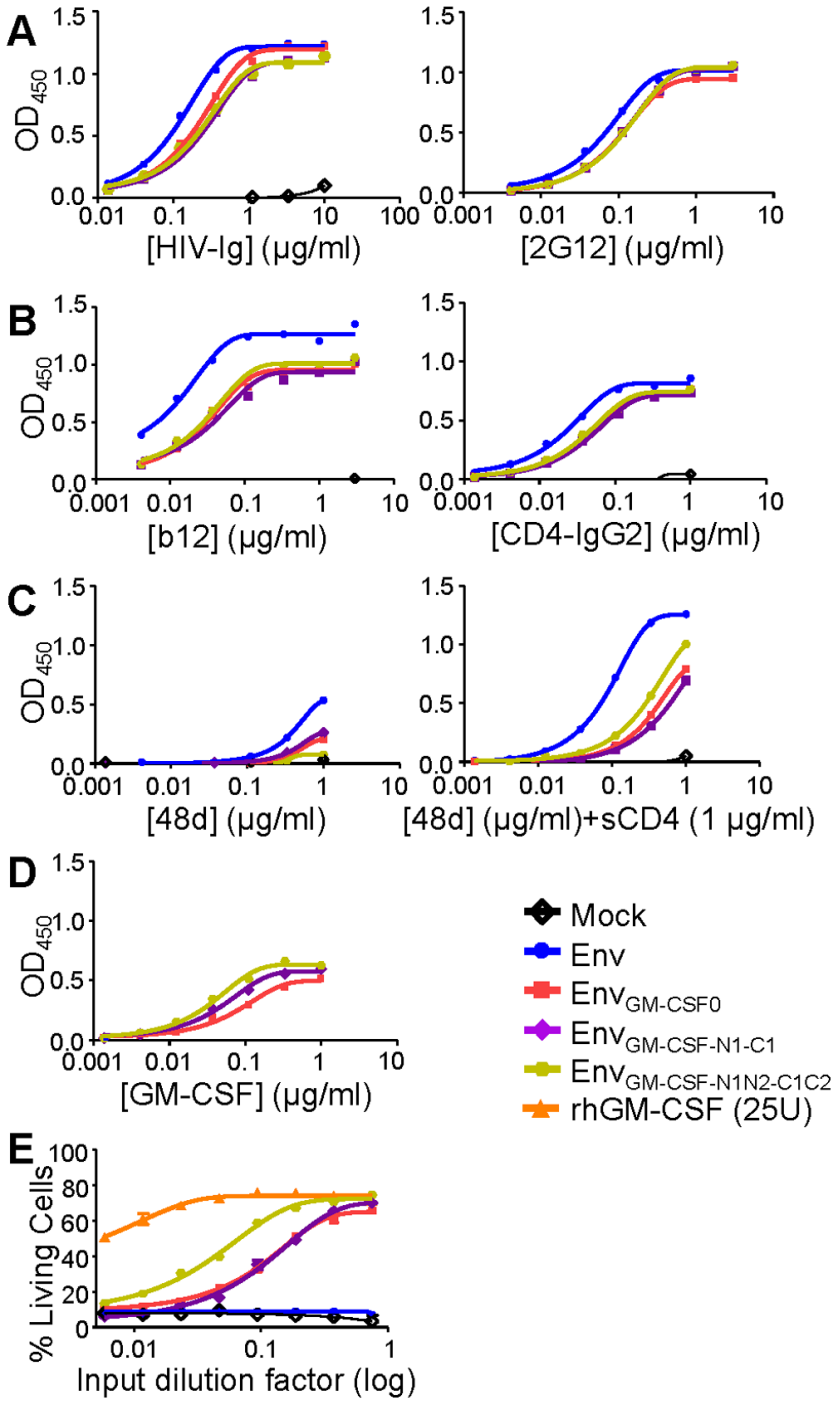
Antigenicity and activity of Env_GM-CSF_ with extended linkers. Recognition of Env_GM-CSF-N1-C1_ and Env_GM-CSF-N1N2-C1C2_ by HIV-Ig and 2G12 (A); b12 and CD4-IgG2 (B); and 48d (CD4i) in the absence and presence of sCD4 (C). Culture supernatant from mock transfected 293T cells was used as a negative control. D. Recognition of Env_GM-CSF-N1-C1_ and Env_GMCSF-N1N2-C1C2_ by GM-CSF antibody (clone 30-4). E. Survival of TF-1 cells in response to culture supernatant containing Env_GM-CSF_, extended linker Env_GM-CSF_ constructs or controls.

Evaluation of the antigenic structure of the extended linker Env_GM-CSF_ constructs showed that the addition of flexible linkers between the Env and GM-CSF domains did not affect the recognition by HIV-Ig and 2G12 (Fig. 8A). Binding of b12 and CD4-IgG2 was similar to the binding to the original Env_GM-CSF0_ (Fig. 8B). Binding of the CD4i mAb 48d in the absence of sCD4 was higher for Env_GM-CSF-N1N2-C1C2_ compared to Env_wt_, Env_GM-CSF0_ and Env_GM-CSF-N1-C1_ (Fig. 8C), indicating that the long flexible linkers allow partial exposure of the CD4i epitope that is normally covered by the V1V2 domain. In the presence of sCD4, Env_GM-CSF-N1N2-C1C2_ bound slightly more strongly to 48d than Env_GM-CSF0_ and Env_GM-CSF-N1-C1_, but not as efficiently as Env_wt_.

We observed increased binding of the anti-GM-CSF mAb (30-4) (ab54429) to Env_GM-CSF-N1N2-C1C2_ compared to Env_GM-CSF0_, indicating that the extension of the linkers improved the accessibility of the GM-CSF domain (Fig. 8D). In agreement with the GM-CSF ELISA data, we observed a 2.6-fold increase in TF-1 cell survival with Env_GM-CSF-N1N2-C1C2_ compared to Env_GM-CSF0_, whereas Env_GM-CSF-N1-C1_ did not show improved GM-CSF activity (Fig. 8E). Collectively, the data shows that extension of the flexible linker regions between the Env and GM-CSF domains improves the accessibility and function of GM-CSF.

### The GM-CSF Domain does not Interfere with Env Cleavage and Vice Versa

**Figure 9:**
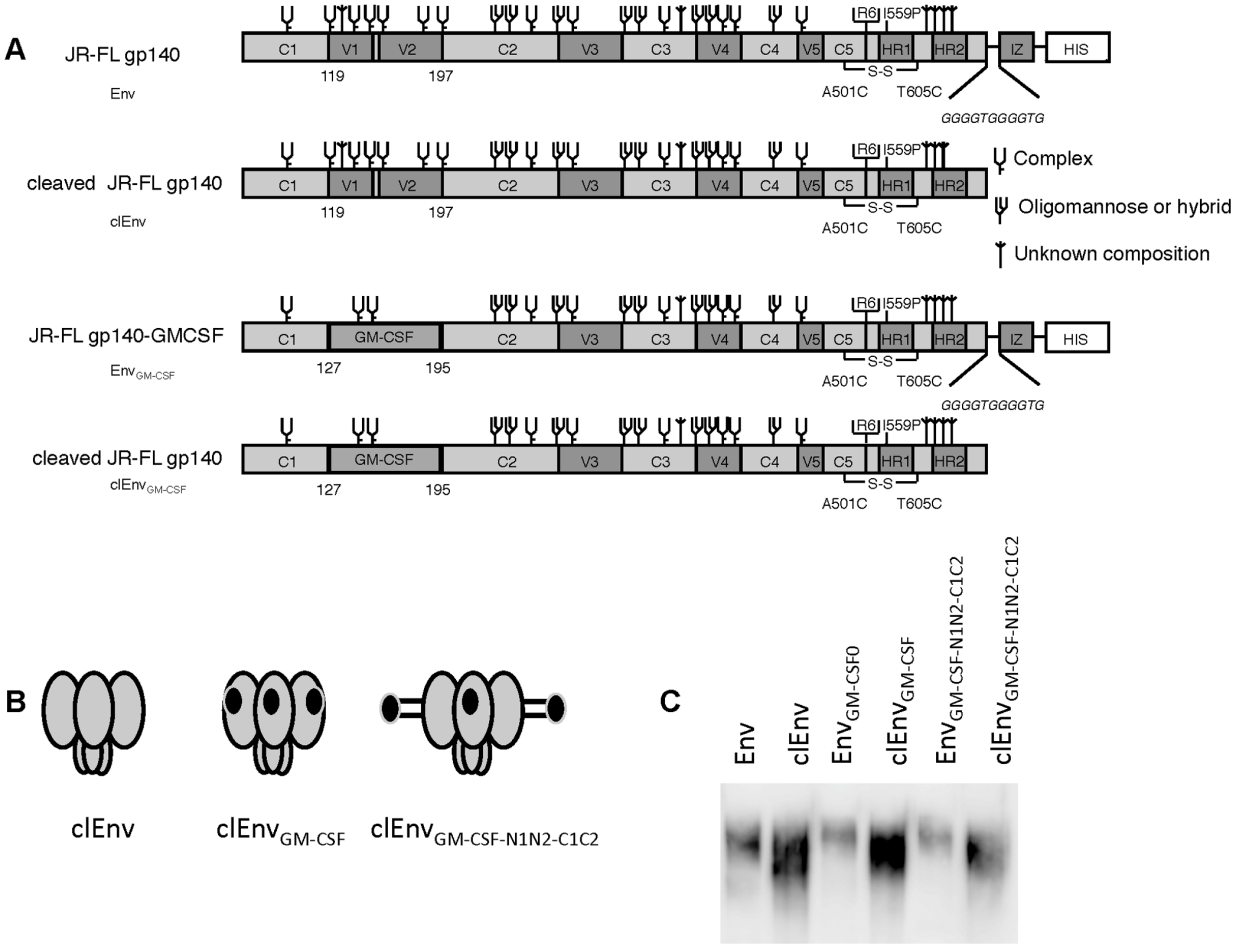
Schematic and expression of the cleaved and linker extended Env_GM-CSF_. Linear (A) and cartoon (B) representation of the Env, clEnv, Env_GM-CSF_ and clEnv_GMCSF_ proteins. Cleaved versions were created by introducing a stop codon in front of the isoleucine zipper (IZ) trimerization domain in the Env_GM-CSF0_, Env_GM-CSF-N1N2-C1C2_ and Env_wt_ constructs. C. Expression of chimeric cleaved Env_GMCSF_ constructs.

Cleavage of the Env precursor gp160 into the gp120 and gp41 subunits is required for Env function. Env cleavage also has major effects on antigenicity. In general BrNAbs tend to bind stronger to cleaved Env, while non-neutralizing Abs favor the uncleaved precursor [16], [18], [32]–[34]. Whether cleavage influences the induction of both types of Abs is not clear. Cleaved soluble trimers are rather instable and dissociate easily. To prevent gp120 dissociation the cleavage site is often mutated and a heterologous trimerization domain is added to form stable Env trimers. In our construct the cleavage site is intact, and gp120 and gp41 are held together by an intermolecular disulfide bond [35]. In addition, we introduced a heterologous trimerization domain to increase trimer stability, but we previously showed that this interfered with cleavage [36]–[38]. To assess the effect of Env cleavage on GM-CSF activity as well as the effect of the embedded GM-CSF on Env’s propensity to become cleaved, we removed the isoleucine zipper (IZ) trimerization domain in the Env_GM-CSF0_, Env_GM-CSF-N1N2-C1C2_ and Env_wt_ constructs (Fig 9A&9B).

The cleavability of the Env trimers was measured by SDS-PAGE under reducing conditions. The cleavable Env (clEnv) and Env_GM-CSF_ variants (clEnv_GM-CSF_) were expressed more efficiently than their uncleaved equivalents and insertion of GM-CSF did not affect the expression, regardless of the linker size (Fig. 9C). Analysis of Env migration on SDS-PAGE under reducing conditions showed that all cleavable Env variants migrated faster through the gel than their non-cleaved counterparts, indicative of gp41 cleavage from gp120 and indicating that replacement of the V1V2 domain by GM-CSF does not interfere with precursor cleavage.

**Figure 10:**
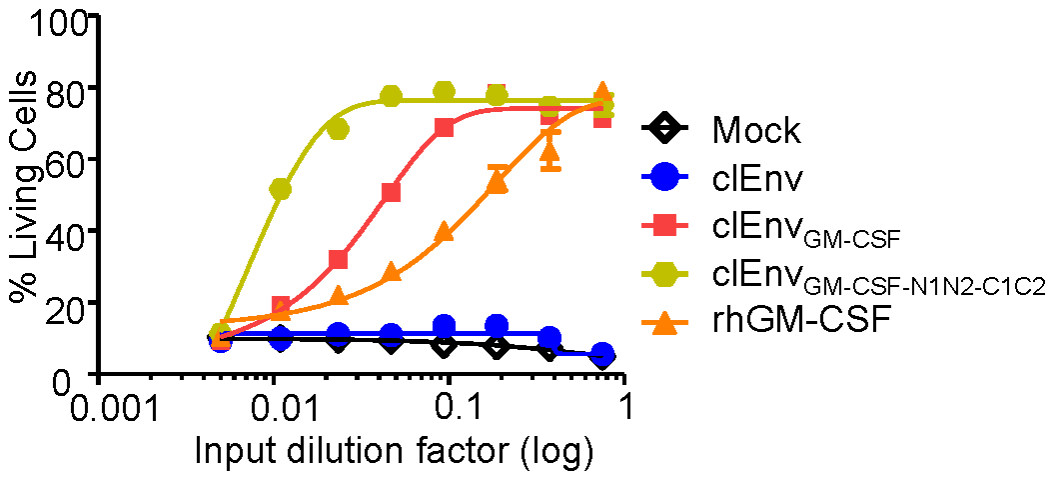
Env cleavage does not interfere with GM-CSF activity. Cell survival of TF-1 cells in response to either culture supernatant containing cleaved Env (clEnv), cleaved Env_GM-CSF_ (clEnv_GM-CSF_), cleaved Env_GM-CSFN1N2-C1C2_ and control reagents.

Next, the effect of Env cleavage on GM-CSF activity was tested on TF-1 cells. We observed that GM-CSF embedded in cleaved Env trimers was active (Fig 10). Extending the linker length for clEnv_GM-CSF-N1N2-C1C2_ resulted in a 5.3-fold higher GM-CSF activity compared to clEnv_GM-CSF_ without additional linkers. Due to the differences in expression of cleaved versus uncleaved constructs, no direct comparison between cleaved and uncleaved Env-GM-CSF constructs could be made, however tested in the same experiment the 5.3-fold increase in GM-CSF activity for cleaved Env trimers caused by the longer linker length was substantially higher than for uncleaved Env trimers (2.6-fold) (Fig. 8E). This may relate to the fact that cleaved trimers are more compact compared to uncleaved trimers [19].

### Chimeric Env_GM-CSF_ Activates Primary Human Monocytes

**Figure 11:**
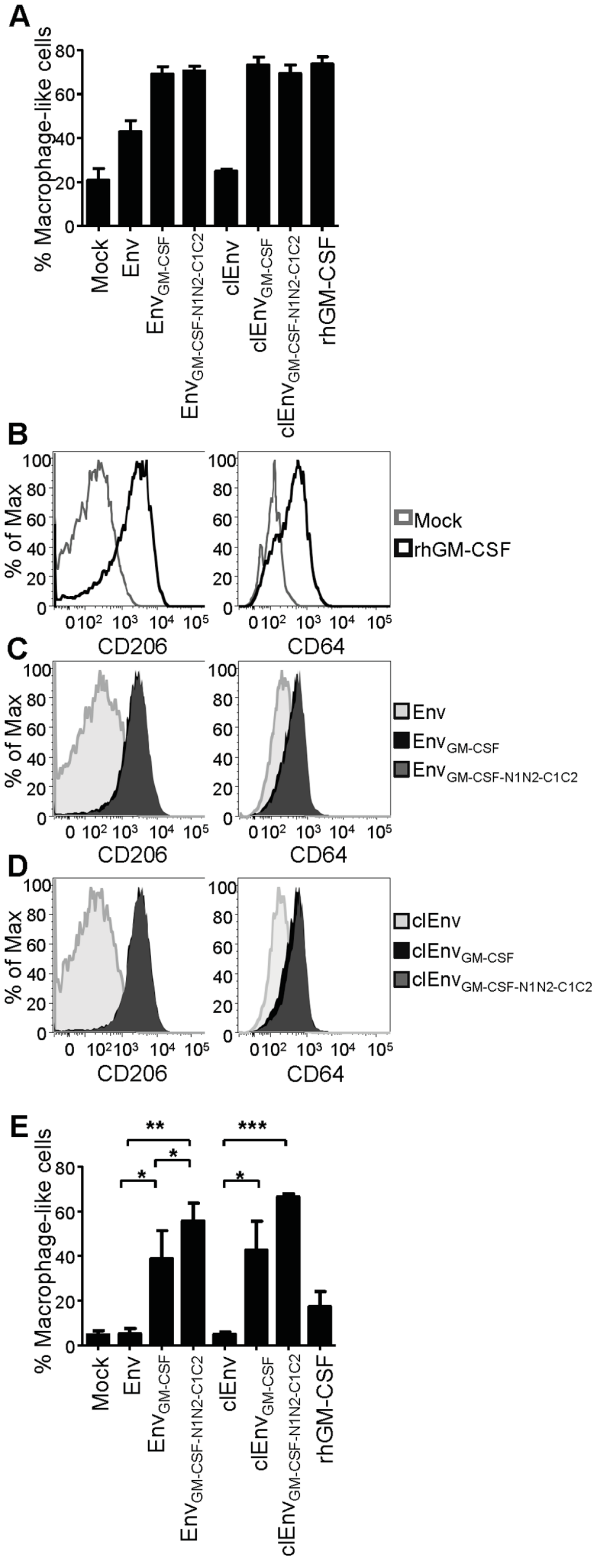
Chimeric Env_GM-CSF_ variants activate primary human monocytes. Monocytes were stimulated with chimeric Env_GM-CSF_ proteins and control and analyzed after 5 days for macrophage-like properties: (A) Percentages of living cells with macrophage-like morphology as analyzed by the SSC vs. FSC in flow cytometry. Data represents the mean of experiments with cells from 2 different donors. Expression of maturation markers CD206 and CD64 on cells incubated with (B) mock (gray line), rhGM-CSF (black line), (C) uncleaved Env (light gray histogram), Env_GM-CSF_ (black filled histogram) and Env_GM-CSF-N1N2-C1C2_ (gray filled histogram), (D) cleaved Env (light gray filled histogram), cleaved Env_GM-CSF_ (black filled histogram) and cleaved Env_GM-CSF-N1N2-C1C2_ (gray filled histogram). Fluorescence intensity (x-axis) is plotted against the percent of Max (y-axis), where the maximum y-axis value in absolute count becomes 100% of total. Data are representative of 5 independent experiments showing similar results. E. Percentages of Macrophage-like cells derived from monocytes deprived of human pooled serum and cultured with fetal calf serum. Data represents the mean of experiments with cells from 3 different donors.

To assess the activity of our Env_GM-CSF_ chimeras on primary human immune cells, we isolated monocytes from human PBMC’s and cultured them with Env, Env_GM-CSF_, Env_GMCSF-N1N2-C1C2,_ clEnv, clEnv_GM-CSF_, clEnv_GMCSF-N1N2-C1C2_ and mock supernatant with or without recombinant GM-CSF. After 5 days of culturing, we performed flow cytometry analysis to assess the differentiation of monocytes towards a macrophage-like phenotype. Based on their morphology (forward/side scatter characteristics), we observed higher percentages of macrophage-like cells in cultures supplemented with Env_GM-CSF_ variants compared to Env, and the levels were similar to these in cultures supplemented with recombinant GM-CSF (Fig. 11A). We also tested the differentiation of monocytes to a macrophage-like phenotype by assessing the expression of the surface markers CD206 (macrophage mannose receptor C) and CD64 (FcγRI) that are characteristics of macrophages [39]–[41]. Cells treated with mock medium had low expression of these markers whereas recombinant GM-CSF increased their expression (Fig.11B). Both uncleaved (Fig.11C) and cleaved Env_GM-CSF_ (Fig.11D) constructs increased the expression of these markers to a similar extent as rhGM-CSF.

In order to test the activity of Env_GM-CSF_ and Env_GM-CSF-N1N2-C1C2_ further, we used more stringent culture conditions in which monocytes were cultured with fetal calf serum instead of human serum, depriving them of essential human serum components. We observed less macrophage-like cells compared to the human serum culture conditions, but the percentage of macrophage-like cells was dramatically elevated with Env containing an embedded GM-CSF compared to Env alone (Fig.11E). In agreement with the TF-1 survival data, extension of the linkers increased the percentage of macrophage-like cells further, both in the context of cleaved and uncleaved chimeric constructs (Env-GM-CSF constructs without versus with extended linkers p = 0.0090). Note that we cannot directly compare the activity of our chimera’s with that of rhGM-CSF because rhGM-CSF is produced in *E. coli* and hence non-glycosylated which affects its function (see above).These results show that our Env_GM-CSF_ constructs can drive the maturation of monocytes into cells with a macrophage-like phenotype.

## Discussion

So far, HIV-1 Env-based subunit vaccines have not been successful in providing immunity against HIV-1. Many intrinsic properties render HIV-1 Env protected against the induction of an adequate antiviral Ab response. Formulating subunit vaccines with adjuvants can improve their immunogenicity. A more sophisticated alternative or complementary approach to simply mixing the antigen and the adjuvant or costimulatory molecule is the direct conjugation or fusion of a co-stimulatory molecule to the antigen. This strategy ensures that the costimulatory molecule activates the exact same immune cells that interact with the antigen. HIV-1 Env has been fused at the N- or C-terminus to costimulatory molecules such as IFN-γ or TNF superfamily members such as APRIL, BAFF and CD40L [37], [38], [42]–[44]. The Env-APRIL fusion construct in particular induced improved neutralizing antibody responses compared to Env alone. As an alternative to N- or C-terminal fusion, we have embedded a co-stimulatory molecule within the Env antigen. In a first prototype chimeric molecule the V1V2 region of Env was replaced with the GM-CSF (Env_GM-CSF_) [23], a pluripotent cytokine acting on different myeloid hematopoietic cells [45], [46], dendritic cells [47], and T-cells [48], leading to enhanced T and B cell responses to Env. A chimeric molecule ideally should have all the preferably properties of both of its components. Here, using a number of protein engineering strategies, we rigorously investigated and optimized these properties for chimeric Env_GM-CSF_, combining optimal GM-CSF activity with an optimal antigenic Env structure and validated these molecules on primary human immune cells.

### Improving GM-CSF Activity of Env_GM-CSF_

Although GM-CSF embedded within Env was functional; it did not have the same activity of soluble rhGM-CSF [23]. We showed that the GM-CSF activity was increased to comparable activity of rhGM-CSF when the linker length between GM-CSF and the Env was extended, as measured by TF-1 cell survival (Fig.8E).

Chimeric Env_GM-CSF_ with an additional six amino acids at both N- and C- termini resulting in 9 amino acids on each side (Env_GM-CSF-N1N2-C1C2_) showed the highest GM-CSF activity. The longer linkers probably provide more conformational and/or steric flexibility that allows better binding to the GM-CSF receptor and formation of dodecameric ligand-receptor complexes. The improved GM-CSF function of Env_GM-CSF-N1N2-C1C2_ may also be attributed to improved exposure of GM-CSF residues 21–31 and 77–94 which have been shown to be critical for GM-CSF activity [49]. In addition to the symmetrical linkers (Fig.7), we also introduced asymmetrical linkers between the Env and GM-CSF and observed that extending the linker at the C-terminal side of GM-CSF is more important for enhancing GM-CSF activity than elongating the linker on the N-terminal side of GM-CSF (data not shown), suggesting that modification of the linkers can affect the orientation of GM-CSF and thereby its activity.

Two *N*-linked glycans are present on GM-CSF, which can influence its systemic half-life, tissue distribution, biological activity and immunogenicity. Bacterially expressed non-glycosylated GM-CSF has been reported to be 10-fold more active than the glycosylated protein. The lower *in vitro* biological activity of glycosylated GM-CSF is thought to be due to reduced affinity for the receptor [31], [50]. Surprisingly however, removal of the two *N*-linked glycans from our Env_GM-CSF_ chimera, did not improve the GM-CSF activity. In fact, removal of the *N*-linked glycan at amino acid position 47 reduced GM-CSF activity dramatically, indicating that this glycan is required for GM-CSF folding and/or function in the context of Env_GM-CSF_. Removing this glycan also reduced the recognition of conformational epitopes on Env, pointing at a global effect on Env_GM-CSF_ folding and/or conformation.

### Improving the Structure of Env in Env_GM-CSF_

An ideal Env antigen should expose most or all epitopes for BrNAbs. Ab binding to most Env epitopes in Env_GM-CSF_ was very similar to that seen with the corresponding, unmodified Env, with the exception of Abs targeting the CD4bs and CD4i epitopes of which the binding was reduced. The extension of the linkers between the Env and GM-CSF subdomains improved the binding of b12 (CD4bs) and 48d (CD4i), suggesting that there were less steric clashes between the GM-CSF subdomain and these Abs.

**Figure 12:**
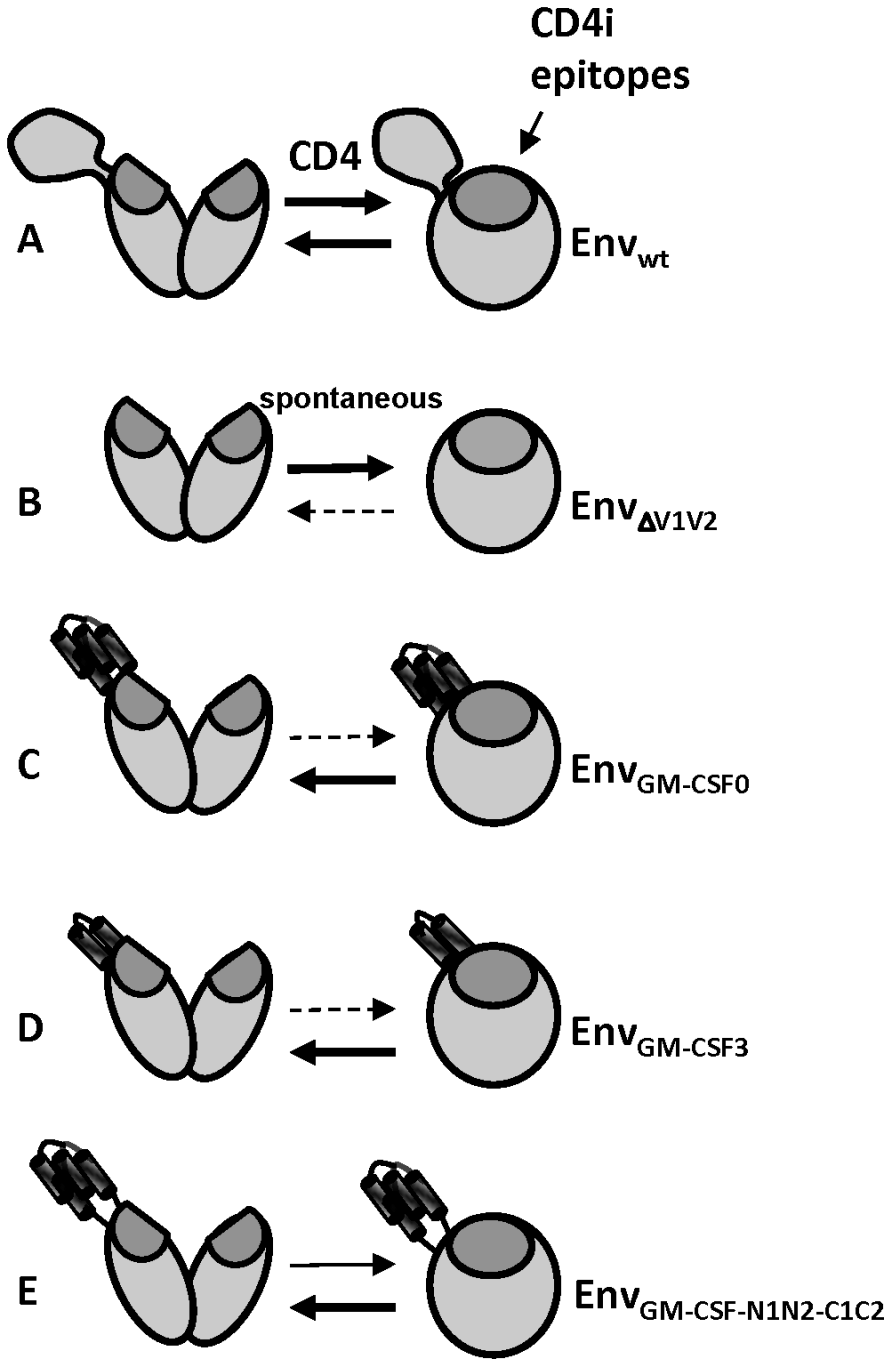
The composition of the V1V2 domain of HIV-1 gp120 modulates the transition between the unliganded and CD4-bound states. Cartoon showing the CD4-induced conformational changes in Env and Env_GM-CSF_ variants. The thickness of the arrows represents direction of the equilibrium. A. Upon CD4 receptor binding wild type Env undergoes conformational changes that involve rearrangement of the V1V2 domain and expose the CD4i epitopes. B. Env lacking the V1V2 domain (Env_ΔV1V2_) folds into the CD4-bound state and exposes CD4i epitopes constitutively. C. When the V1V2 domain is replaced with GM-CSF, the CD4i antibodies cannot bind, irrespective of the presence of CD4, indicating that intrinsic properties of the V1V2 allow the CD4-induced changes. D. Replacement of GM-CSF with smaller GM-CSF variants that were more similar in size to the V1V2, did not improve the exposure of the CD4i, suggesting that the size of the V1V2 is not a critical factor for allowing CD4-induced conformational changes to occur. E. The addition of flexible linkers between the Env and GM-CSF domains slightly improved the binding of CD4-IgG2 and CD4i antibodies.

In wt Env, binding of CD4 induces conformational changes that involve repositioning of the V1V2 domain, thereby exposing the CD4i epitopes that can be targeted by antibodies such as 48d (Fig.12A) [51]. In contrast, when the V1V2 domain is deleted, Env tends to fold spontaneously into a conformation that exposes the CD4i epitopes [3], [52]. Thus, the presence of the V1V2 prevents the formation of premature folding into the CD4i state, but also allows for such conformational changes to occur once CD4 has bound (Fig.12B). Replacement of the V1V2 domain with GM-CSF resulted in a protein that was not recognized by CD4i mAbs in the absence of CD4, suggesting that the presence of bulk protein grafted onto the V1V2 stem can block premature folding into the CD4i state. However, Env_GM-CSF_ was also not efficiently recognized by 48d when CD4 was added, indicating that the presence of GM-CSF in the place of V1V2 blocks the CD4-induced conformation changes, and indicating that properties intrinsic to the V1V2 allow the CD4-induced changes (Fig. 12). Replacement of GM-CSF with smaller GM-CSF variants that were more similar in size to the V1V2, did not improve the exposure of the CD4i epitope, suggesting that the size of the V1V2 is not a decisive factor in allowing CD4-induced conformational changes to occur (Fig. 12). GM-CSF and the V1V2 are structurally quite different (GM-CSF is a four-helix bundle [53], while the V1V2 domain is folded into a four stranded anti-parallel β-sheet which is stabilized by disulfide bonds and from which the V1 and V2 loops emanate [8]), but it is unclear what specific V1V2 properties allow for the conformational changes upon CD4 binding.

An ideal Env antigen should probably be a structural mimic of the unliganded state, not the CD4-bound state, since the unliganded Env spike is the target of BrNAbs and Abs such as 48d that target the CD4i epitopes are only weakly neutralizing because their epitopes become only exposed after the virus binds to CD4, at which point the space and time for antibodies to block viral entry is limited [54]. The fact that replacing the V1V2 domain by GM-CSF rigidifies Env and blocks conformational changes down the entry pathway may therefore improve the stability of the native unliganded state and favor the induction of BrNAbs.

In our view, an ideal Env antigen should be cleaved between gp120 and gp41, because the native Env spike on the virus is cleaved. Cleavage of precursor gp160 into the gp120 and gp41 subunits is required for Env function. Furthermore, uncleaved Env and cleaved Env are antigenically distinct and in general, BrNAbs favor cleaved Env trimers while non-neutralizing Abs favor uncleaved Env [19]–[22]. Therefore, we designed cleavable Env_GM-CSF_ variants and found that Env cleavage did not interfere with the functionality of GM-CSF embedded within the V1V2 domain, nor did the presence of GM-CSF affect Env cleavage.

### Conclusions

Our chimeric Env_GM-CSF_ proteins have many desirable properties and combine a favorable Env structure with the immunostimulatory activity of GM-CSF. We plan to test the immunogenicity of the Env_GM-CSF-N1N2-C1C2_ construct in comparison with Env_GM-CSF_ in rabbits. We favor, rabbits over mice for such experiments because rabbits are able to produce antibodies with long CDRH3 domains that are commonly found in neutralizing HIV antibodies [55]. Of course chimeric Env_GM-CSF_ is not a perfect mimic of the Env spike on virus. For example, the quaternary structure dependent BrNAbs PG9 and PG16 [56]–[58] are not able to bind to Env_GM-CSF_, because their epitopes require conserved elements within the V1V2 domain. The correlates and sieve analyses of the modestly successful RV144 trial showed that Abs directed to the V1V2 domain correlated with lower infection rates among vaccinees and that vaccinees were partially protected against viruses with particular signatures in the V2 [59]. Thus, removing this region in Env_GM-CSF_ may eliminate potentially protective epitopes. It remains to be seen whether improving overall Env immunogenicity by the insertion of GM-CSF outweighs the loss of V1V2 epitopes.

Other cytokines have a similar four-helix bundle structure as GM-CSF and this allows us to design other chimeric Env_cytokine_ proteins with immune stimulating properties (“envokines”). Indeed we have successfully created Env_IL-2_, Env_IL-4_ and Env_IL-21_ envokines by replacing the GM-CSF domain by these cytokines in the V1V2 domain. In particular the Env_IL-21_ envokine is very potent in activating human B cells, which may be a desirable property for inducing a potent Env-directed B cell response. Furthermore, in parallel, we are currently investigating whether we can insert GM-CSF into other locations than the V1V2 domain to test the activity and antigenicity of the chimeric molecules side by side. In conclusion, we engineered chimeric Env-cytokine molecules that potently activate human immune cells yet retain an antigenic Env structure that should be favorable for inducing humoral immunity against HIV-1.

## Methods

### Ethics Statement

*N/A.*

### Constructs

The plasmid expressing codon-optimized stabilized HIV-1 gp140 (SOSIP.R6-IZ) was used as the starting point for the constructs generated in this study (Fig. 1A). The Env is based on the subtype B, CCR5-using primary isolate JR-FL and is described in detail in elsewhere [3], [19], [32], [35], [36]. Amino acid numbering is based on HXB2 gp160 according to convention. Codon-optimized DNA encoding human granulocyte macrophage colony-stimulating factor (GM-CSF) flanked by HindIII and BmgBI restriction sites was synthesized (Mr. Gene Regensburg, Germany) and the V1V2 domain of Env was exchanged with the sequences coding for GM-CSF using the HindIII and BmgBI sites. Substitutions and insertions were generated using the QuikChange™ mutagenesis kit (Stratagene, CA, USA). The sequence integrity of all constructs was verified by sequencing.

### Reagents

HIV-Ig was obtained through the AIDS Research and Reference Reagent Program (ARRRP), Division of AIDS, NIAID, NIH. MAb 2G12 was obtained from Hermann Katinger through the ARRRP. CD4-IgG2, sCD4 and anti-V3 gp120 MAb PA1 were gifts from Bill Olson (Progenics Pharmaceutical, Tarrytown, NY). MAb b12 was donated by Dennis Burton (The Scripps Research Institute, La Jolla, CA). MAb 48d was a gift from James Robinson (Tulane University, New Orleans, LA). The mouse monoclonal GM-CSF antibody (clone 30-4, ab54429) was purchased from Abcam (Cambridge, UK). Recombinant human GM-CSF (rhGM-CSF) for TF-1 cell stimulation was obtained from Schering-Plough (Brussels, Belgium).

### Cell Lines

293T cells were maintained in Dulbecco’s Modified Eagle’s Medium (DMEM; Invitrogen, Breda, The Netherlands) supplemented with 10% heat inactivated fetal calf serum (FCS; HyClone, Perbio, Etten-Leur, the Netherlands), MEM nonessential amino acids (0.1 mM; Invitrogen, Breda, the Netherlands) and penicillin/streptomycin (both at 100 U/ml). TF-1 cells [23], a gift from Paul Coffer, were cultured in RPMI 1640 (Invitrogen) with 10% FCS, supplemented with 25 U/ml GM-CSF (Schering-Plough, Brussels, Belgium).

### Transfections

293T cells were transiently transfected with plasmids expressing recombinant Env using linear polyethylenimine (PEI, MW 25,000; Polysciences Europe GmbH, Eppelheim, Germany) as described elsewhere [23]. Briefly, plasmid DNA was diluted in 1/10 of the final culture volume of DMEM and mixed with PEI (0.15 mg/ml final concentration). After incubation for 20 min, the DNA-PEI mix was added to the cells for 4 h before replacement with regular culture medium. Env containing supernatants were harvested 48 h after transfection and frozen in aliquots.

### Trimer ELISA

Env trimer ELISA was performed as previously described [3], [36]. Briefly, supernatants containing His-tagged Env gp140 proteins were diluted 1∶3 in TBS (10 mM Tris, 150 mM NaCl, pH 7.5) supplemented with 10% FCS and added for 2 h to pre-blocked Ni-NTA HisSorb 96-well plates (Qiagen, Venlo, The Netherlands). After three washes using TSM (20 mM Tris, 150 mM NaCl, 1 mM CaCl_2_, and 2 mM MgCl_2_), serially diluted polyclonal HIV-Ig, Env specific monoclonal Abs, or CD4-IgG2, in TSM 5% BSA was then added for 2 h, with or without 1 µg/ml sCD4, followed by three washes with TSM, 0.05% Tween20. Horseradish peroxidase (HRP)-labeled goat-anti-human immunoglobulin G (0.2 µg/ml, Jackson Immunoresearch, Suffolk, UK) was used as secondary Ab and the absorption at 450 nm was measured after the colorimetric reaction was stopped using H_2_SO_4_. Equal input levels were verified SDS-PAGE followed by western blot analysis.

### SDS-PAGE and Western Blotting

SDS-polyacrylamide gel electrophoresis (SDS-PAGE) and western blotting were performed as previously described [36]. Env was detected using the MAb PA1 (0.2 µg/ml) and a 1∶5,000 diluted secondary HRP-labeled goat-anti-mouse IgG (Kirkegaard & Perry Laboratories, Maryland, USA) followed by detection using the Western Lightning ECL solution (PerkinElmer, Groningen, The Netherlands).

### GM-CSF Activity Assay

Myeloid leukaemia TF-1 cells (5.0×10^4^ in 50 µl) were seeded in a 96-well plate. Serially diluted supernatants from Env_wt_, Env_GM-CSF_ variants, mock transfected 293T cells, or rhGM-CSF diluted in supernatant from mock transfected 293T cells were added to the cells. Equal input levels were verified SDS-PAGE followed by western blot analysis. Each sample was tested in quadruplicate wells. The cells were harvested on day five and the percentage of living cells was determined by flow cytometry analysis.

### Stimulation of Monocytes

Peripheral blood mononuclear cells (PBMCs) were isolated from buffy coats of healthy blood donors using Lymphoprep (Axis-Shield, Oslo, Norway) density gradient centrifugation. Monocytes were isolated by adherence to plastic and cultured in Iscove’s modified Dulbecco’s medium (IMDM; Lonza, Basel, Switzerland) supplemented with 10% (v/v) heat-inactivated human pooled serum (HPS) or 10% (v/v) heat inactivated fetal calf serum (FCS; HyClone, Perbio, Etten-Leur, The Netherlands), penicillin (100 U/ml; Invitrogen, Carlsbad, CA), streptomycin (100 µg/ml; Invitrogen) and ciproxin (5 µg/ml; Bayer, Leverkusen, Germany) for 5 days in the presence of Env constructs produced from 293T cells, GM-CSF (50 ng/ml; Peprotech London, UK), or medium alone at 37°C in a humidified atmosphere supplemented with 5% CO_2_.

### Flow Cytometry

Cells were fixed with 1% paraformaldehyde for 10 min at room temperature and subsequently washed with PBS containing 0.5% BSA, 10% 0.13 M trisodiumcitrate, 2 mM EDTA and 0.01% Na-azide. Cells were stained with the following antibodies for 30 min at 4°C in the dark: anti-human conjugated to CD16-Alexa Fluor 647 (AbD Serotec, MorphoSys, Oxford, UK), CD64-Alexa Fluor 488 (Biolegend, San Diego, CA, USA), CD80-PE-Cy5 (BD Biosciences, San Jose, CA, USA), CD163-PE (BD Biosciences), CD206-APC-Cy7 (Biolegend). Mouse IgG1κ conjugated to FITC, PE, PerCP-Cy5.5, APC and APC-Cy7 was used as isotype control (BD Biosciences). Flow cytometry was performed with the FacsCanto II (BD Biosciences) and the results were analyzed in FlowJo, version 9.4.3 (Tree Star, Ashland, OR, USA).

### Statistical Analysis

Percentages of macrophage-like cells were assessed by analyzing the SSC vs. FSC plot in flow cytometry. The statistical significance of differences was tested using a paired student’s T-Test (GraphPad Prism 5).
